# Chitosan–Hydroxycinnamic Acids Conjugates: Emerging Biomaterials with Rising Applications in Biomedicine

**DOI:** 10.3390/ijms232012473

**Published:** 2022-10-18

**Authors:** Doddy Denise Ojeda-Hernández, Alejandro A. Canales-Aguirre, Jordi A. Matias-Guiu, Jorge Matias-Guiu, Ulises Gómez-Pinedo, Juan Carlos Mateos-Díaz

**Affiliations:** 1 Laboratory of Neurobiology, Institute of Neurosciences, IdISSC, Hospital Clínico San Carlos, Universidad Complutense de Madrid, 28040 Madrid, Spain; 2 Preclinical Evaluation Unit, Medical and Pharmaceutical Biotechnology Unit, CIATEJ-CONACyT, Guadalajara 44270, Mexico; 3 Department of Neurology, Institute of Neurosciences, IdISSC, Hospital Clínico San Carlos, Universidad Complutense de Madrid, 28040 Madrid, Spain; 4 Department of Industrial Biotechnology, CIATEJ-CONACyT, Zapopan 45019, Mexico

**Keywords:** chitosan, hydroxycinnamic acids, biomaterials, biomedical applications, conjugates, chitosan derivatives, phenolics

## Abstract

Over the past thirty years, research has shown the huge potential of chitosan in biomedical applications such as drug delivery, tissue engineering and regeneration, cancer therapy, and antimicrobial treatments, among others. One of the major advantages of this interesting polysaccharide is its modifiability, which facilitates its use in tailor-made applications. In this way, the molecular structure of chitosan has been conjugated with multiple molecules to modify its mechanical, biological, or chemical properties. Here, we review the conjugation of chitosan with some bioactive molecules: hydroxycinnamic acids (HCAs); since these derivatives have been probed to enhance some of the biological effects of chitosan and to fine-tune its characteristics for its application in the biomedical field. First, the main characteristics of chitosan and HCAs are presented; then, the currently employed conjugation strategies between chitosan and HCAs are described; and, finally, the studied biomedical applications of these derivatives are discussed to present their limitations and advantages, which could lead to proximal therapeutic uses.

## 1. Introduction

Over the years, molecules of natural origin, such as chitosan, have become an object of interest for the development of novel drugs and biomaterials. Natural-origin molecules can display active properties and can also be useful ingredients for the elaboration of biocompatible and stable formulations for novel therapeutic technologies [1,2].

Chitosan (CS) is a natural-origin amino-polysaccharide mainly composed of D-glucosamine and, in a lower proportion, N-acetyl-D-glucosamine units that are randomly β-(1–4)-linked. CS has been used in different biomedical applications, mainly as a drug carrier, wound accelerator, hemostatic agent, fat binder, antimicrobial agent, and recently, as a bio-ink ingredient for 3D printing and bioprinting [3,4]. The increasing interest in this polymer has led to the search for strategies that expand its applications. In this way, the molecular structure of this polymer has offered an opportunity to add specific mechanical, chemical, or biological characteristics through its conjugation with other molecules [5]. Functionalization of CS is possible since it possesses functional groups (amino and hydroxy) that are free for binding with other interesting bioactive molecules. However, the final characteristics of the functionalized products are defined by multiple factors that remain a research subject, such as the molecule to be attached to the binding sites, the degree of functionalization, and the intrinsic characteristics of the starting CS [6]. Therefore, researchers have focused on exploring new substituents with defined properties that suit different applications, such as hydroxycinnamic acids (HCAs). HCAs have gained attention due to their interesting biological activities and health benefits [7]. Moreover, their carboxylic group can be easily attached to the aforementioned functional groups that are present in the polymeric chains of CS. As will be further described, HCAs can provide CS with new functions and mechanical properties that increase its applicability, and can also potentiate the CS intrinsic biological activities through a synergistic effect, unlike other CS derivatives (e.g., CS acetate, CS sulfate). CS–HCA derivatives have shown promising applications in the biomedical field, where they have been employed for tissue engineering, biomolecule delivery, protection against infections, and the treatment of other specific conditions.

In this review article, the main attributes and disadvantages of CS and HCAs are described, the currently employed conjugation strategies are discussed, then the characteristics of the CS–HCA derivatives that have been studied for biomedical applications to date are detailed, and finally, potential future research developments in the area are presented.

## 2. Chitosan

**Figure 1:**
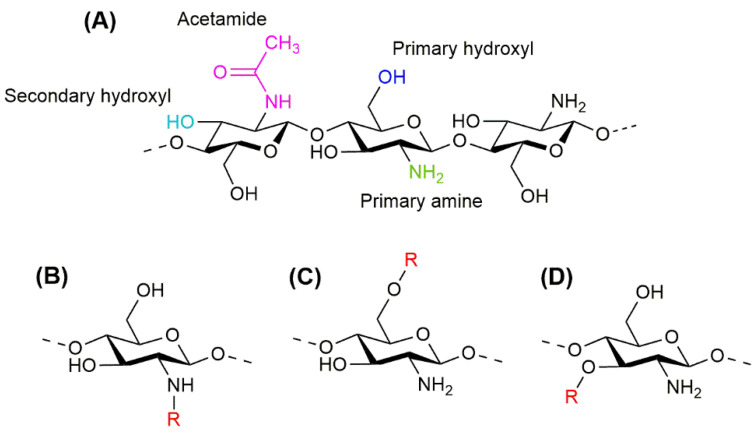
Molecular structure of chitosan (**A**), indicating its functional groups, primary amine (green), primary hydroxyl (blue), secondary hydroxyl (light blue), and acetamide (magenta); and the molecular structure of CS derivatives with substitutions at the amino group (**B**), the primary hydroxyl group (**C**), and the secondary hydroxyl group (**D**), indicating the substituents as R (red).

CS is an amino-polysaccharide whose monomeric units are N-acetyl-D-glucosamine and, predominantly, D-glucosamine, which are connected by β-(1–4)-glycosidic linkages. The molecular structure of CS contains an amino/acetamido group at C-2, a secondary hydroxyl group at C-3, and a primary hydroxyl group at C6 (Figure 1A). The amine function on the CS structure provides it with a polycationic nature, and jointly with the hydroxyl groups, makes CS subject to chemical modifications.

CS is usually obtained from the deacetylation of chitin. Chitin is one of the most abundant polysaccharides in nature and is composed of the same monomeric units as CS, but, in contrast to CS, N-acetyl-D-glucosamine is present in a higher proportion than D-glucosamine. Nearly 70% of chitin comes from marine species, but it is also present in diverse organisms such as sea animals, insects, fungi, and microorganisms [8]. Currently, the main commercial sources of chitin and CS are shrimp and crab shell waste. The industrial production of CS from these wastes requires the implementation of chemical processes for the demineralization, discoloration, deproteinization, and deacetylation of chitin [9]. Inconveniently, raw materials for chitin extraction tend to be seasonal and variable according to each species. Moreover, variations in the chitin deacetylation process have been shown to cause an effect on some of the properties of CS-based final products [10]. As a result, different fungal sources have been proposed for the obtention of CS, since the physical properties of the extractable CS can be manipulated through culturing techniques and crude fungal chitin does not require the demineralization step [9,11]. In this way, CS from fungal sources can be obtained by employing milder conditions and generating less hazardous chemical wastes than shellfish sources. Moreover, it can have defined properties and is free of the concerns about shellfish allergen proteins and heavy metals content, which makes it attractive for healthcare uses [12]. Although fungal sources are not as abundant as crustacean wastes, it has been suggested that the fungal culture for different purposes could be a possible source of biomass for CS obtention [13]. In addition to the search for new sources, alternatives to the chemical obtention of CS, such as microbiological or enzymatic methods, have also been suggested. Fermentation-based techniques employ seafood wastes as substrates for the growth of different microorganisms, achieving a substantial reduction of proteins and minerals present in the raw material [14,15,16]. Enzymatic treatments employ proteases, chitin deacetylases, chitinases, chitosanases, and chitooligosaccharide deacetylases to obtain CS. Chitin and CS-modifying enzymes enable the possibility to obtain CS oligosaccharides with defined architecture, but enzymatic modifications get complicated when the molecular weight (MW) and the crystallinity of CS are high, due to the limited solubility of the polymer and the enzyme’s difficulty of access [17,18]. Therefore, the aforementioned biological methods require additional chemical processing, either as a pretreatment or to refine the characteristics of the product. As a result, it has been suggested that unconventional chemical methods, such as the enhancement of the reactions by microwave or ultrasound, could be implemented for more eco-friendly processes [19].

Depending on the desired application for the CS, its characteristics must be carefully selected, giving great importance to its deacetylation degree (DD), MW, and purity, since they can affect the mechanical and biological properties of the final product. In a review article, Farhadihosseinabadi et al. discussed the interactions of CS with different cell types, where the effects of the MW, crystallinity, and DD of CS over cell interaction are also considered [20]. For example, the authors assert that high DD values are associated with a decreased biodegradation rate, and the MW of CS modulates the viscosity affecting its interaction with the surrounding tissue [20].

Beyond the selection of the source and the intrinsic characteristics of CS, the functionalization, i.e., the modification of CS molecular structure by its conjugation with other molecules, can rationally change the properties of CS-based bioproducts. It is important to mention that the nature of the substituent, the binding site, and the functionalization degree deeply affect the CS derivatives’ behaviors [21]. Mainly, the functionalizing substituents have been grafted to the CS structure at its primary amino group (Figure 1B) due to its higher reactivity, but it has been reported that the amine function of CS has a strong influence over its biological activities, as antimicrobial activity, wound healing, mucoadhesiveness or macromolecules adhesion, and stem cell proliferation and differentiation [22,23,24,25]. Therefore, the functionalization degree can be modulated to keep some of the amine function properties, or different strategies can be implemented to bind the substituents at the hydroxyl groups (Figure 1C,D). In this way, many types of molecules have been employed for the functionalization of CS, such as proteins and peptides, natural and synthetic polymers, and ceramics, among others [26,27,28,29].

Given the interesting properties of CS, including its modifiable structure, the research focus is turning towards predesigned modifications to precisely modulate the characteristics of CS-based products. Here, the subject focuses on a group of interesting molecules (i.e., HCAs) employed for the functionalization of CS, as further described in the following section.

## 3. Hydroxycinnamic Acids

**Figure 2:**
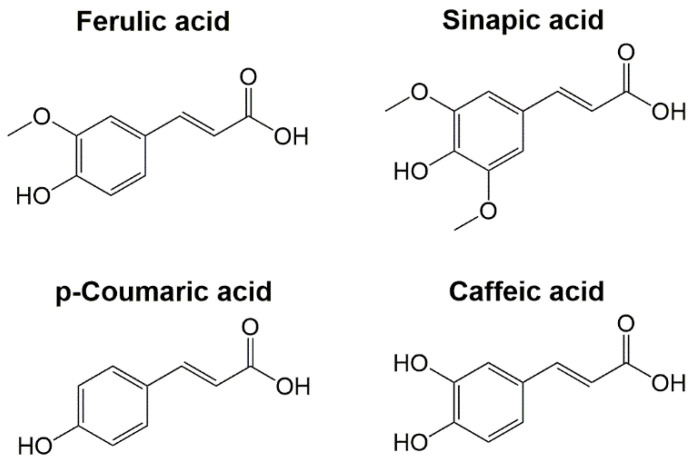
Molecular structure of most common hydroxycinnamic acids, i.e., ferulic acid, sinapic acid, p-coumaric acid, and caffeic acid.

Phenolic compounds are plant secondary metabolites that possess many health benefits, e.g., their dietary consumption has been related to the prevention of cognitive disorders, cardiovascular disease, metabolic syndrome, cancer, and diabetes [30,31]. Their natural origin coupled with their interesting bioactivities have made them one of the most studied molecules by the scientific community [32]. Phenolic acids are one of the main classes of plant phenolic compounds synthesized from phenylalanine and tyrosine in the shikimate and phenylpropanoid pathways. There are two major groups of phenolic acids: hydroxybenzoic acids, with a C6–C1 structure, and HCAs, with a C6–C3 structure. The most cited examples of HCAs include ferulic acid (FA), sinapic acid (SA), p-coumaric acid (pCA), and caffeic acid (CA). Their molecular structure contains hydroxyl and methoxyl groups on the phenolic ring and a carboxylic group in the C3 lateral chain (Figure 2). The functional groups (hydroxyl and methoxyl) in the HCAs molecular structures provide them with an interesting antioxidant activity. Moreover, the presence of an ethylene chain contributes to a strong H-donating ability and radical stabilization [33]. HCAs are naturally found in fruits, vegetables, and cereals; however, they are mostly linked to hydroxylic acids, saccharides, flavonoids, or other plant structural biopolymers, such as cellulose, proteins, and lignin, to form esters [7,34]. Therefore, different chemical methods to obtain HCAs have been employed, such as the use of alkaline hydrolysis followed by organic solvent extraction [35,36]. More eco-friendly processes that use industrial wastes as departing raw materials to obtain HCAs have been implemented [37,38,39]. In the same way, different enzymes such as pectinases, pentosanases, and mainly feruloyl esterases have been used for the enzymatic hydrolysis and further extraction of HCAs [40,41,42]. 

HCAs have been studied, either on their own or in combination with other molecules, for biomedical applications. They have exhibited antioxidant, anticancer, anti-inflammatory, antimicrobial, antidiabetic, and neuroprotective properties, among others [34,43,44]. The sources, characteristics, and bioactivities of each HCA will be detailed below. 

The importance of understanding the structure–activity relationship in potential biomedical applications is heightened since the use of grafted HCAs has been recently extended in this field. In this way, it is important to understand the biological function of the molecular structure of HCAs, so it can be kept or modified consciously when conjugated with other molecules.

### 3.1. Ferulic Acid

FA is the most abundant HCA, and it is mainly found in whole grains, spinach, parsley, and grapes. In a review article, Kumar and Pruthi showed the ferulic acid content in different known sources [45], and found that the higher values were found in sugar beet pulp, corn, and bamboo shoots. However, many of the processes that have been proposed for its obtention employ wheat, maize, or rice brans as raw materials, since they are abundant agricultural by-products [46,47,48]. 

The chemical structure of FA contains a hydroxyl and a methoxyl group at the para- and meta-position of the aromatic ring, respectively. The presence of the methoxyl group enhances the electron-donating function provided by the hydroxyl group. This structural arrangement confers in FA a strong antioxidant activity through ROS removal and the regulation of oxidative stress-related enzymes [49,50,51,52]. The antioxidant activity of FA has been solidly associated with its photoprotective, regeneration and wound healing, anti-inflammatory, antifibrosis, neuroprotective, and cell-protection properties [51,53,54,55,56]. Mostly, the relation between the antioxidant activity of FA and its bioactivities is associated with the effects of oxidative stress on age-related diseases [57]. FA has also shown anti-Alzheimer’s disease activity and behavioral impairment improvement capacity, antiplacental inflammation and apoptosis, therapeutic potential in peripheral arterial disease, attenuation of adipocyte differentiation, protection against metabolic syndrome, anticancer activity, and neuroprotective effects against cerebral ischemia, among other properties for biomedical applications [58,59,60,61,62,63,64].

### 3.2. Sinapic Acid

SA is present in spices, vegetables, citrus, berries, and mainly in cereals [65]. Its obtention from food manufacturing by-products has been recently proposed, using mustard bran, rapeseed, and canola extracts as starting materials [66,67]. The molecular structure of SA contains one hydroxyl group and two methoxyl groups at the para- and meta-position of the aromatic ring, respectively. The incorporation of another methoxyl group in comparison with FA confers in SA an increased antioxidant activity and a decreased redox potential [68]. In this way, SA has been reported to possess DPPH, superoxide anion, hydroxyl, hydroperoxyl, hypochlorite, nitric oxide, and peroxynitrite radicals scavenging activity [69]. Additionally, protection against lipid peroxidation and free-radical-mediated diseases have been reported [70,71]. Some of the studied bioactivities of SA are protection against hypertension-associated cardiac dysfunction, and anticancer, bone regeneration, neuroprotection, and anti-inflammatory properties [72,73,74,75,76], among other protective potentials recently reviewed by Pandi et al. [77].

### 3.3. p-Coumaric Acid

pCA contains only one substituent in the aromatic ring, a hydroxyl group at the para-position. As mentioned, the presence of a hydroxyl group at the para-position of the phenolic ring has been related to antioxidant activity. Although there are other isomers of coumaric acid, i.e., ortho- and meta-, the para-isomer is the most abundant in nature [78]. pCA can be obtained from fruits, vegetables, mushrooms, plant products, and cereals, and it is also present in beverages such as red wine [79]. Its content is not as abundant as FA, so many of the proposed strategies for pCA obtention employ grains and their by-products, green propolis, and grasses [80,81,82]. 

Many health benefits have been attributed to pCA, such as antiangiogenic effects, protection of lens epithelial cells, protective effects on ethanol-induced male reproductive toxicity, protection against diabetes-associated spontaneous destruction of periodontal tissue, anti-inflammatory effects in rheumatoid arthritis, ameliorating of ionizing radiation-induced intestinal injury, anti-inflammatory effects in acute lung injury, neuroprotection, and protection against high-fat diet-induced metabolic dysregulation [83,84,85,86,87,88,89,90,91]. The main bioactivities of pCA have also been related to its antioxidant properties through the blocking of ROS accumulation, but there is still a need for more studies to elucidate the activity in other possible metabolic pathways.

### 3.4. Caffeic Acid

CA is probably the most studied of the HCAs, followed by FA. CA is the most abundant HCA in fruits, so it can be found in berries, cherries, apples, kiwi, and quince, among others. It can be also found in vegetables, beverages, olive oil, and many medicinal plants [7,92,93]. Although it is believed that coffee is a major source of CA human intake, the novel approaches for its extraction consider herbs/plants and the use of methods assisted by ultrasonic, supercritical fluids, and molecularly imprinted polymers [94,95,96,97].

An interesting feature of the molecular structure of CA is the incorporation of two hydroxyl substituents at the phenolic ring (Figure 2), leading to a significant decrease in the redox potential and thus increasing the antioxidant activity. It is due to the important antioxidant properties of CA that it has been studied to find its potential for multiple applications. In the health field, it has been observed that the antioxidant and anti-inflammatory activities of CA provide protection against different oxidative and degenerative processes. In this way, CA protects against oxidative/nitrosative damage, hepatic and renal dysfunction in aflatoxicosis, cartilage degradation in osteoarthritis, and modulates mechanisms related to intestinal inflammation [98,99,100]. The vasorelaxant activity and antiangiogenic effects of CA contribute to protecting against atherosclerosis. Additionally, it possesses immunomodulatory properties, antimicrobial activity against human pathogenic bacteria, protection against endometriosis progression, and neuroprotective effects as it reduces the oxidative stress and microglial activation in the hippocampus, preventing neurodegeneration in mice [101,102,103,104,105,106]. CA has been recently proposed as a potential modulator of oncogenic molecular pathways since it can induce apoptosis in cancer cells, take part in cell mechanisms associated with cancer progression in cancer therapy, sensitize cancer cells to chemotherapy, and suppress the proliferation of HeLa cells [107,108].

The long list of the compelling bioactivities of HCAs keeps the application potential of these molecules in the spotlight of biomedical research. However, structure–activity studies are in the minority, and a long way remains to understand their implication in biological processes, particularly since their conjugation with other molecules (e.g., biopolymers) has shown to increase their applicability [109].

## 4. Conjugation of Chitosan and Hydroxycinnamic Acids

**Figure 3:**
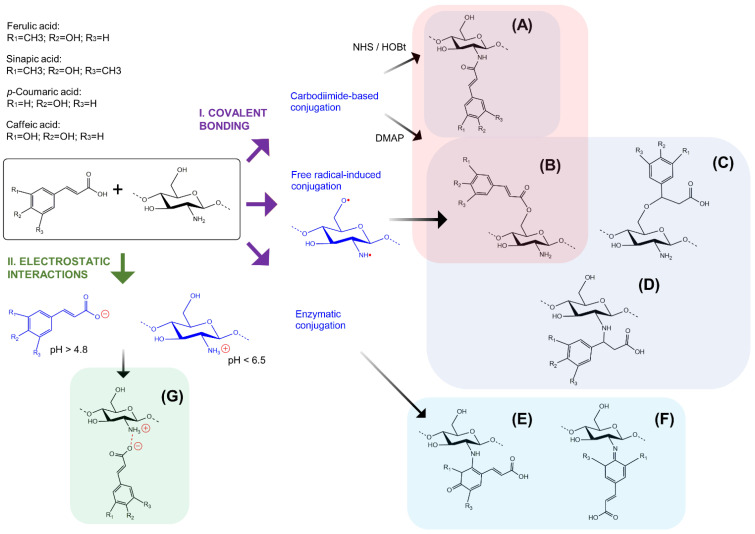
Conjugation of chitosan and hydroxycinnamic acids. **I.** Most employed conjugation strategies to obtain covalently bonded CS–HCAs. Carbodiimide-based conjugation employs mostly dicyclohexylcarbodiimide (DCC) or diisopropylcarbodiimide (DIC) or 1-(3-dimethylaminopropyl)-3-ethylcarbodiimide (EDC) as coupling agents and 1-hydroxybenzotriazole (HOBt) or N-hydroxysuccinimide (NHS) as nucleophilic additives (**A**). Sometimes 4-(Dimethylamino)pyridine (DMAP) is employed as an acylation catalyst, which is crucial for the efficient formation of esters (**B**). Free radical-induced conjugation uses ascorbic acid and hydrogen peroxide to produce hydroxyl and ascorbate free radicals which capture hydrogen atoms from -OH, and -NH_2_ chitosan groups, forming chitosan radicals that react with HCAs to form CS–HCA conjugates (**A**–**D**). Enzymatic conjugation uses different polyphenol oxidases to oxidize HCAs into phenoxyl radicals, which are converted into quinones, and in turn, couples covalently with NH_2_ moieties of chitosan through Michael addition (**E**) or imine formation (Shiff base) (**F**). **II.** Most common non-covalent conjugation to obtain CS–HCAs through electrostatic interactions. The carboxyl group of HCAs can deprotonate at pH values above its pKa (≈4.8), giving place to negative-charged polar groups that interact electrostatically with positively charged amino groups of CS at pH values below its pKa (≈6.5) (**G**).

Due to the multiple beneficial properties of CS and HCAs, scientists have focused on the different strategies to use them together, taking advantage of the structural characteristics and biological activities of both. In this way, the obtention of HCA-grafted CS (CS–HCAs) can be achieved by different general conjugation strategies, which have been described in detail by some authors and can be consulted elsewhere [28,109,110,111,112]. Here, a brief description of the main conjugation strategies to obtain CS–HCA conjugates is presented (Figure 3).

### 4.1. Covalent Bonding

Most of the conjugation strategies employed to graft HCAs to the CS structure make use of covalent bonding (Figure 3I). It is known that conjugated products can achieve a higher stability through covalent bonds, although other non-covalent interactions are involved too [113]. Chemical methods have been successfully employed for this purpose, but the necessity of carrying out greener processes with reduced environmental impacts and toxicity potential has led to the search for enzymatic alternatives.

#### 4.1.1. Chemical Methods

Different coupling agents have been used as linkers to assist in the conjugation between CS amine or hydroxyl groups and HCAs, such as the notable case of carbodiimides (Figure 3A,B). Carbodiimide-based methods are the most employed for CS–HCA synthesis, being 1-ethyl-3-(3-dimethylaminopropyl)carbodiimide (EDC) the preferred coupling agent due to its high solubility in water. EDC reacts first with carboxylic groups of HCAs, forming an O-acylisourea intermediate with a similar reactivity to the corresponding carboxylic acid anhydride, which further reacts with the primary amino groups of CS and couples HCAs to CS through an amide bond (Figure 1B and Figure 3A). Additives, such as N-hydroxysuccinimide (NHS) or 1-hydroxybenzotriazol (HOBt), are commonly employed together with EDC, as nucleophilic additives, to form a more stable and activated intermediate ester to react with amine groups of CS [114]. For the efficient formation of esters (Figure 3B), the addition of EDC (or any carbodiimide, e.g., DCC or DIC) and 4-(Dimethylamino)pyridine (DMAP) as a catalyst is crucial, as previously reported [115]. One of the advantages of using EDC as a coupling agent is that urea, formed as a by-product after CS–HCA synthesis, is water-soluble, which facilitates the purification process [116]. Moreover, EDC-based reactions can be carried out under mild conditions, preventing the degradation of the CS polymeric chains. EDC-assisted reactions are commonly performed under acidic conditions to facilitate CS solubility. However, it is known that these reactions are pH-sensitive. It has been suggested that the pKa values of amino groups can be used to optimize the amidation [117], but it has also been reported that the use of cosolvents, such as dimethyl sulfoxide (DMSO), can cause changes in the apparent pKa value of the reagents, changing their properties in the reaction [118]. Therefore, the starting pH value and nature and amount of cosolvents, along with other parameters such as temperature, the ratio of molar equivalents, MW and DD of CS, and reaction time, should be considered when planning EDC-based reaction conditions. The influence of these many factors on the successful synthesis of CS–HCAs has led to high variability in the yield of the conjugation reactions, also influencing the properties of the resulting products. Nevertheless, the conjugation of HCAs and CS consistently enhances the resulting products’ bioactivities [119,120]. 

A conjugation strategy that does not require the use of coupling agents has also been popular for CS–HCA synthesis: free radical-induced conjugation. The ascorbic acid/hydrogen peroxide redox pair has been the most used for this conjugation strategy, where the reaction mechanism has been proposed by different authors [110,111]. In brief, the ascorbic acid reacts with hydrogen peroxide and forms hydroxyl and ascorbate free radicals. Then, the hydroxyl radical abstracts hydrogen from the CS amino and hydroxyl groups, giving place to a CS radical. HCAs can be acceptors of the CS radical and give place to CS–HCA conjugates (Figure 3A–D). An inert atmosphere, such as nitrogen, is often employed to achieve this reaction since it protects the HCAs from oxidation and increases the grafting degree. Additionally, it has been observed that the grafting degree is influenced by the concentration of the reagents, reaction time, pH, and temperature, as well as the presence of oxygen. The products obtained by this conjugation strategy include CS–HCAs through the bonding of primary amino or hydroxyl group from CS with either the carboxylic or alkene group from HCAs [121,122]. In addition, when Ceric (IV) ammonium nitrate is employed as a redox initiator, the CS ring opens due to the formation of a complex of the oxidizing agent with the primary amine and the hydroxyl group at the C-3 position of CS, which is followed by the formation of radicals by the complex dissociation [123]. Although free radical-induced conjugation is a more eco-friendly and mild-conditioned strategy, a careful selection of redox reagents must be conducted to achieve the desired molecular structure for the conjugates.

The conversion of the functional groups in CS has become another strategy for its coupling with HCAs. For this purpose, the amino or hydroxyl groups in CS can be substituted with different components, followed by a grafting reaction with HCAs. For example, triphenylphosphine and N-bromosuccinimide have been used to replace the primary hydroxyl group at C-6 of CS with bromine. Then, C-6-bromine could further react with ethylenediamine to synthesize a C-6-aminoethyl group that is able to bind with the HCA [124]. In this way, it is possible to use as starting materials some CS derivatives where amino groups are already substituted, such as trimethylated chitosan. Additionally, modification of HCAs has been proposed in a novel strategy that was implemented to optimize the synthesis of CS–HCAs, where tert-butyldimethylsilyl-protected HCAs were converted to acyl chlorides and reacted with 3,6-di-O-tert-butyldimethylsilyl-chitosan for their conjugation through amide-bonding [125].

#### 4.1.2. Enzymatic Methods

Enzymatic modifications have gained attention, since they reduce the utilization of polluting compounds, employ mild conditions, and offer high specificity and selectivity. Different enzymes have been used for this purpose, such as laccases, peroxidases, and tyrosinases. Laccases have been the most explored enzymes for grafting phenolics to CS, as they are considered safe and simply available. As a result, these enzymes have gained attention for biomedical and pharmaceutical applications [126]. As reviewed by Shokri et al., laccases employed for the conjugation between CS and phenolics have been obtained from different fungal and bacterial strains, such as *Myceliophtora thermophyla*, *Bacillus vallismortis*, *Trametes versicolor*, and *Bacillus* sp. PC-3 [127]. The laccase-mediated conjugation occurs through the oxidation of hydroxyl groups born by the phenolic ring of HCAs (Figure 3E,F), which has put in doubt the preservation of the biological properties granted by HCAs. Nevertheless, it has been reported that the enzymatic grafting of CA on CS has shown to be more functionally active than unmodified CS or even than the mixture of CA and CS [128]. In this way, the laccase-mediated functionalization of biopolymers with phenolics, and particularly with FA, has been shown to give place to hydrogels and other interesting biomaterials with tailor-made properties suitable for biomedical applications [129]. In addition to laccases, tyrosinases have been employed for grafting HCAs to CS (i.e., CA), showing advantages for the grafting of phenols bearing a single hydroxyl group [130]. Moreover, other enzymes have been employed for the grafting of phenolics to CS and could eventually be used for grafting HCAs. Such is the case of the horseradish peroxidase that has been used to graft gallic acid to CS, where the grafting occurred between the amino group of CS and the carboxyl group of gallic acid [131].

### 4.2. Non-Covalent Interactions

The combination of CS and HCAs gives place to non-covalent interactions, i.e., hydrogen bonds and electrostatic interactions. Docking simulation has been employed for the understanding of the interaction between HCAs and CS, and it has been found that FA and SA form three hydrogen bonds each, while CA forms two hydrogen bonds. In addition, it was observed that both moieties were involved in hydrogen bonding, phenolic oxygen and carboxylic acid [132]. Hydrogen bonds are often present in CS hydrogels as well, where these bridges form intermolecular networks [133]. Electrostatic interactions mainly occur between the negative charges of the substituents and positively charged groups in CS. In this way, the carboxyl group of HCAs can deprotonate at pH values above its pKa (≈4.8), giving place to negative-charged polar groups that interact electrostatically with positively charged amino groups of CS at pH values below its pKa (≈6.5) (Figure 3G) [134]. These interactions can allow improved physicomechanical strength in biomaterials, but with little control over the association and interaction patterns [112,133]. In this way, the conjugation between CS and HCAs through non-covalent interactions has been shown to reduce cytotoxicity, improve the mechanical properties, and enhance the thermal stability of the products [76].

As previously described, the different conjugation strategies entail advantages and disadvantages that may need to be considered for each intended application. Additionally, the standardisation of the manufacturing processes will be necessary for large-scale production, where it is important to keep in mind the environmental commitment and the increasing tendency to employ non-hazardous reagents.

## 5. Biomedical Applications of CS–HCAs Conjugates

In the biomedical field, CS derivatives have been mainly used for skin, bone, neural, and vascular tissue engineering, dental applications, drug delivery, and as antimicrobial agents. Various review articles detail the properties and composition of the CS derivatives that have been evaluated according to each biomedical application [135,136,137,138,139,140,141,142]. There, the authors have discussed the mechanical, chemical, and biological properties of the CS derivatives, intending to correlate them with their composition and contribute to the understanding of their interdependence.

**Figure 4:**
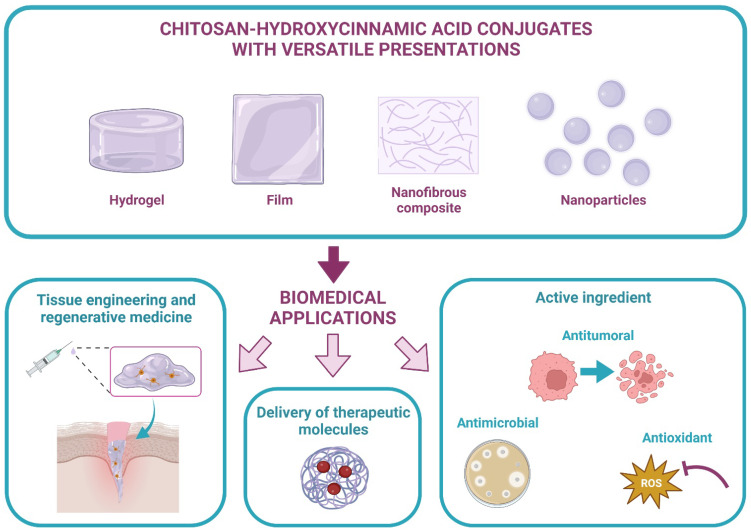
Versatile presentations of CS–HCAs and their reported biomedical applications.

Likewise, CS–HCA biomaterials possess a high versatility that grants them the capacity to acquire different presentations. Hydrogels, films, nanofibrous composites, and nanoparticles have been obtained for biomedical applications. Mainly, three broad applications of CS–HCA conjugates are being employed currently: the delivery of therapeutic molecules, repair and tissue engineering, and as an active ingredient (Figure 4). These applications will be detailed further. It is important to mention that most of the reported studies implement covalent bonds for the conjugation between CS and HCAs, so it is only specified here in cases where ionic bonding is used.

### 5.1. Delivery of Therapeutic Molecules

The four HCAs have been employed to conjugate CS with delivery purposes, where FA is the most used among them. CS–HCA conjugates can be used as suitable carriers due to their capacity for nano- and micro- encapsulating therapeutic molecules. Moreover, the previously mentioned bioactivities of CS and HCAs can provide these carriers with new biological properties or enhance the already expected ones. Although most of the evaluations have been made employing model molecules, they exhibit promising results as a proof of concept.

In this way, Li et al. have prepared CS-FA conjugates via an ascorbic acid-hydrogen peroxide redox pair system and by a carbodiimide-mediated coupling reaction. The obtained products showed, in both cases, a reduced crystallinity and higher thermostability. Furthermore, encapsulation using the spray drying technique with bovine serum albumin, as a proof of concept, has exhibited a controlled release and swelling capacity in comparison with CS alone [143,144]. The use of CS-FA conjugate made by a free radical-mediated grafting method to micro-encapsulate thiamine and pyridoxine has displayed a good encapsulation rate and controlled release, also showing an anti-inflammatory potential. This additional property was found in vivo over carrageenan-induced paw oedema in albino rats, which was indicatively attributed to a synergistic effect between the therapeutic molecules and the FA from the conjugate [145]. 

Kamal et al. encapsulated *Syzygium aromaticum* essential oil in a CS-pCA nanogel and found that the chemotherapeutic effects were boosted, compared to the unencapsulated oil. There, the antimicrobial activity was tested over *Escherichia coli* and *Staphylococcus aureus*, and in vitro anticancer activity was evaluated over the breast (MCF-7) and skin (A-375) cancer cell lines. It is worth noting that the reported results for those activities indicated higher values for the CS-pCA biomaterial than the essential oil [146]. A CS derivative, thiolated CS, has also been employed for the obtention of a pCA conjugate, showing an increased swelling capacity and mucoadhesion. The controlled release of a hydrophobic molecule, piperine, was achieved when homocysteine thiolactone was attached to thiolated CS-pCA conjugates [147].

CS-CA conjugated via carbodiimide coupling has been used for its copolymerization with dextran-*b*-poly(ethylene glycol) for nano-encapsulating doxorubicin and its evaluation over doxorubicin-resistant CT26 cells. There, growth inhibition was found and attributed to the entry of the nanoparticles into the cells, a condition that was not observed for non-encapsulated doxorubicin treatment [148].

Huber et al. compared the enzymatic conjugation of CS with trans-FA, CA, or SA for the obtention of hydrogels. Good in vitro biocompatibility was observed for CS-trans-FA and -CA conjugates over immortalized human embryonic kidney cells (ATCC HEK293), but not for CS-SA. In that work, the methylene blue release capacity proved to be dependent on the substrate employed for CS conjugating. CS–HCA hydrogels were found to be swelling-controlled systems, where a higher concentration of CS was related to a higher swelling capacity and thus, to the increase of the releasing properties [149]. 

The use of CS–HCAs in the delivery of therapeutic molecules is resumed in Table 1.

### 5.2. Tissue Engineering and Regenerative Medicine

Biomaterials employed for tissue engineering and regenerative medicine require specific physical and chemical properties, in such a manner that they mimic the structural and compositional characteristics of the target tissue [150]. Therefore, the reported research for these applications combines CS–HCAs with other biopolymers, or employs CS derivatives as starting material to obtain defined mechanical properties. It is worth noting that, in the bibliographic search carried out for the writing of this review article, no reports were found employing SA to conjugate CS for this application (Table 2). 

QS-HCA conjugates have been mainly employed for one of the most studied applications of regenerative medicine: wound healing, where biocompatibility and antimicrobial properties play a fundamental role. For this purpose, poly(ɛ-caprolactone) (PɛC) has been combined with CS-CA to obtain a micro-fibrous material for wound dressing. The grafting of CA to CS showed an increase in the tensile properties, the attachment and proliferation of human dermal fibroblast-neonatal (NHDF-neo) cells, and antimicrobial activity again *S. aureus* [151]. Similarly, PɛC has been combined with CS-FA (ionic bonding) to elaborate a nano-fibrous material. The grafting of FA to either CS or PɛC also increased the antimicrobial activity against *S. aureus*, but higher antitumor effects were only found for CS-FA against human cervical tumor cells (HeLa). These results suggested an additional potential application for the local delivery of antitumor drugs [152]. Moreover, the incorporation of FA into CS has been shown to enhance the water-absorbing capacity and the antimicrobial activity of CS against *S. aureus* and *E. coli* when combined with bacterial cellulose [153]. In the same way, the incorporation of pCA-CS (ionic bonded) into a polyvinyl alcohol/starch film has been shown to provide antimicrobial activity over *S. aureus* [154]. When combined with gelatin, the addition of FA to CS (ionic bonded) for the elaboration of films increased the water uptake, strength, and heat resistance in a solvent-dependent manner [155]. A CS derivative, O-carboxymethyl CS, has been non-covalently conjugated with CA for the elaboration of a composite with polyacrylamide. The obtained composite was evaluated as a doxycycline-carrying hydrogel for wound dressing. Results indicated a controlled delivery of doxycycline, non-toxicity, and growth inhibition of *S. aureus* and *E. coli* [156]. Another CS derivative, glycol CS, was used by Wei et al. for its conjugation with FA. Then, glycol CS-FA conjugate was enzymatically crosslinked with feruloyl-modified peptides to elaborate antioxidant hydrogels for cutaneous wound healing. The in vivo evaluation in a full-thick skin defect model showed an accelerated wound closure process and the formation of mature skin through promoting fibroblast migration and re-epithelization [157]. Aljawish and Muniglia enzymatically grafted FA to CS for the elaboration of films for tissue engineering. The incorporation of FA increased the hydrophobic and antioxidant properties of the films and promoted a higher viability and adhesion of the mesenchymal stem cells (MSCs) when compared to CS alone or its conjugation with the FA ethyl-ester (i.e., ethyl ferulate) [158].

CS–HCAs have also shown potential therapeutic applications for neural tissue repair. Glycol CS-FA has been synthesized by chemical means for the elaboration of nanoparticles that effectively deliver both CS and FA to the injury site in a model of spinal cord injury. There, the grafting of FA showed in vitro to prevent neuronal loss and neurite disintegration caused by glutamate-induced excitotoxicity. Additionally, glycol CS-FA nanoparticles in vivo preserved axons and myelin, and reduced activated astrocytes, macrophages, and the cavity size at the lesion site, leading to functional recovery to a greater extent compared to CS alone [159]. A FA and succinic acid-grafted CS (CS-FA/SuA) has been employed for the elaboration of hydrogels and evaluated over a traumatic brain injury (TBI) model. CS-FA/SuA has shown to possess good in vitro biocompatibility over MSCs and mouse fibroblast (L929) cells, and also displays good cell adhesion over expanded allogeneic adipose-derived stem cells (eASCs). After its intracerebral administration in a TBI model, CS-FA/SuA hydrogel ameliorated the brain damage by reducing the activation of astrocytes and microglia when small amounts of FA were employed for the preparation of the conjugates, preventing secondary injury as well [115].

The use of CS–HCAs in tissue engineering and regenerative medicine is resumed in Table 2.

**Table 2:** Chitosan-hydroxycinnamic acid conjugates in tissue engineering and regenerative medicine.

Conjugate	Synthesis Method	Application	Study Type	Properties	Reference (Year)
CS-FA combined with bacterial cellulose	Ascorbic acid/hydrogen peroxide redox pair	Films for wound healing	In vitro	Enhanced water-absorbing capacity and antimicrobial activity over *S. aureus* and *E. coli*	Shen et al. (2021) [153]
CS-pCA combined with polyvinyl alcohol/starch films	Non-covalent interactions	Nanoparticles or films for wound healing	In vitro	CS-pCA film with higher antioxidant activity and less cytotoxicity over L929 cells CS-pCA nanoparticles with better thermal stability and enhanced antimicrobial activity over Gram-positive and Gram-negative bacteria	Lee et al. (2021) [154]
CS-FA/ Succinic acid	Carbodiimide-mediated	Hydrogels for brain injury repair	In vitro/in vivo	Enhanced in vitro biocompatibility with MSC and L929 cells, and cell-adhesion capacity of eASC In vivo integration to neural tissue and biocompatibility	Ojeda-Hernández et al. (2021) [115]
O-carboxymethyl-CS-CA combined with polyacrylamide	Non-covalent interactions	Doxycycline carrying hydrogel for wound dressing	In vitro	Controlled delivery of doxycycline, non-toxicity over HDF cells, and growth inhibition of *S. aureus* and *E. coli*	Hafezi et al. (2020) [156]
Glycol-CS-FA crosslinked with feruloyl-modified peptides	Carbodiimide-mediated conjugation and enzymatic (laccase) crosslinking	Wound healing hydrogels	In vitro/in vivo	Higher storage and compressive modulus Enhanced in vitro antioxidant activity and biocompatibility with NIH-3T3 cells In vivo acceleration of wound closure process and promotion of mature skin formation	Wei et al. (2019) [157]
CS-FA combined with PɛC	Carbodiimide-mediated	Nanofibrous material for wound dressing and for local treatment of cervical tumours	In vitro	Higher viscosity and swelling capacity, enhanced antibacterial activity against *S. aureus*, and higher antitumor activity over HeLa cells	Yakub et al. (2018) [152]
CS-FA combined with gelatin	Non-covalent interactions	Films/scaffolds for biomedical applications	In vitro	Formation of a polymer coacervation system, higher water uptake, and porosity when formic acid was used as the solvent	Nady et al. (2018) [155]
CS-FA	Enzymatic (laccase)	Biomaterials for tissue engineering and other biomedical applications	In vitro	Higher hydrophobic and antioxidant properties, and enhanced viability and cell-adhesion of MSC	Aljawish et al. (2016) [158]
CS-CA combined with PɛC	Ascorbic acid/hydrogen peroxide redox pair	Microfibrous material for wound dressing	In vitro	Increased tensile properties, higher cell attachment and proliferation of NHDF-neo cells, enhanced antimicrobial effect against *S. aureus*	Oh et al. (2016) [151]
Glycol CS-FA	Carbodiimide-mediated	Nanoparticles for functional restoration of traumatically injured spinal cord	In vitro/in vivo	In vitro protection of primary neurons from glutamate-induced excitotoxicity In vivo preservation of axons and myelin, and cavity volume, astrogliosis, and inflammatory response reduction at the spinal cord contusion injury site	Wu et al. (2014) [159]

CS, chitosan; FA, ferulic acid; pCA, p-coumaric acid; CA, caffeic acid; PɛC, poly(ɛ-caprolactone); HDF, human dermal fibroblast; NHDF-neo, normal human dermal fibroblast-neonatal cells; MSC, mesenchymal stem cells; eASC, expanded adipose stem cells.

### 5.3. QS-HCAs as Active Ingredients

#### 5.3.1. Antimicrobial

CS–HCA conjugates have been reported as antimicrobial agents in recent years, gaining interest in food preservation and biomedical applications [111,160]. The biomedical field has taken advantage of the antimicrobial effects of these conjugates mainly for wound dressing, as previously mentioned. However, a potential synergistic effect has been suggested, not only between CS and HCAs but also between CS–HCAs and antibiotics, leading to an increasing interest in its use as an active ingredient.

Lee et al. compared the antimicrobial effect of the covalent grafting of FA, SA, or CA onto the CS backbone. The evaluation of 15 clinical isolates and two standard methicillin-resistant *S. aureus* strains, three standard methicillin-susceptible *S. aureus* strains, and eight foodborne pathogens showed higher inhibitory activity from the conjugates than the unmodified CS. Although the higher antimicrobial activity was obtained from CS-FA, the highest DPPH scavenging activity was obtained from the CS-CA conjugate, and the highest lipid peroxidation inhibitory activity was obtained from the CS-SA. Additionally, there was reported a good biocompatibility of the evaluated CS–HCAs on human Chang liver and mouse macrophage RAW264.7 cells [161]. The CS-FA conjugate has shown bactericidal and antibiofilm potential as well. These effects have been attributed to the alteration of the cell membrane integrity and permeability on human pathogenic bacteria (*Listeria monocytogenes*, *Pseudomonas aeruginosa*, and *S. aureus*), and to the coaction of FA combined with the CS capability to eradicate mature biofilms [162]. 

The synergic effect of CS–HCAs with antibiotics has been evaluated by Kim et al., where the incorporation of CA to CS enhanced the antibacterial activity, to a greater extent than FA, SA, or unmodified CS, against acne-related bacteria (*S. aureus*, *Staphylococcus epidermidis*, *Pseudomonas aeruginosa*, and *Propionibacterium acnes*). CS-CA also showed a synergistic antibacterial effect when combined with commercial antibiotics used to treat *acne vulgaris* (tetracycline, erythromycin, and lincomycin) [163]. In the same way, the grafting of FA to CS has been shown to increase the antimicrobial effect when combined with β-lactam antibiotics (ampicillin, penicillin, and oxacillin) against methicillin-resistant *S. aureus*. There, a restoration of the susceptibility of the bacterial strain to those antibiotics was suggested through the inhibition of the expression of methicillin resistance-associated genes [164].

#### 5.3.2. Antitumoral

Although the antitumoral properties of CS–HCAs have been shown to be useful in antitumor drug delivery, these conjugates have also been suggested as anticancer compounds (Table 3). In this way, FA, SA, and pCA have been employed to conjugate CS quaternary ammonium derivatives. The resulting antioxidant and antitumoral activities were higher compared to the unmodified CS quaternary ammonium derivatives. The antioxidant activity pattern of the conjugated products followed the order: SA > FA > pCA, while the antitumor effects against lung cancer cells (A549) seemed to be superior when FA or SA was grafted to CS. Additionally, the evaluated CS–HCA showed good biocompatibility over L929 cells [124]. Likewise, the conjugation of CS with CA has increased the antitumoral activity compared with unmodified CS over colorectal carcinoma cells (CT26), where an anti-invasive effect has also been observed against Matrigel-cultured cells [165].

#### 5.3.3. Antioxidant

The antioxidant capacity of CS–HCAs has been related to many of the properties that give great potential to these conjugates in the already mentioned biomedical applications. In addition, the use of CS–HCAs as antioxidant agents has also been demonstrated in vivo (Table 3). For that purpose, Liu et al. covalently linked FA or CA to CS, showing that treatment employing those conjugates not only decreased lipid peroxidation in vivo, but also increased the activity of antioxidant enzymes (superoxide dismutase, glutathione peroxidase, and catalase) over a D-galactose induced ageing mice model. Both in vitro and in vivo results showed slightly higher antioxidant activity from the CS-CA in comparison to CS-FA, and a significant enhancement from both conjugates compared to unmodified CS [166]. Additionally, the antioxidant potential of CA-grafted CS has been shown to provide a hepatoprotective effect against oxidative stress-induced hepatic damage in ethanol-induced liver injury in mice. There, the conjugate significantly increased the activities of superoxide dismutase, catalase, and glutathione peroxidase, and down-regulated the TNF-α and IL-6 gene expressions in the liver [122].

The use of CS–HCAs as active ingredients is resumed in Table 3.

**Table 3:** Chitosan-hydroxycinnamic acid conjugates as active ingredients.

Conjugate	Synthesis Method	Application	Study Type	Properties	Reference (Year)
CS-FA	Ascorbic acid/hydrogen peroxide redox pair	Biomaterial with antibacterial activity	In vitro	Bactericidal action against *L. monocytogenes* and *S. aureus*, and bacteriostatic action against *P. aeruginosa*	Dasagrandhi et al. (2018) [162]
CS-CA, CS-FA, CS-SA	Ascorbic acid/hydrogen peroxide redox pair	Antibacterial agents to control antibiotic- resistant acne-related bacteria	In vitro	Enhanced antimicrobial activity, especially from CS-CA Reduction of MIC values of antibiotics against antibiotic-resistant *P. acnes* and *P. aeruginosa*	Kim et al. (2017) [163]
CS-FA	Ascorbic acid/hydrogen peroxide redox pair	Antibacterial agent to use in combination with antibiotics against methicillin-resistant *S. aureus*	In vitro	Higher antioxidant activity and restoration of susceptibility of methicillin-resistant *S. aureus* to β-lactams	Eom et al. (2016) [164]
CS-FA, CS-CA, CS-SA	Ascorbic acid/hydrogen peroxide redox pair	Antibacterial agent against methicillin-resistant/susceptible *S. aureus* and foodborne pathogens	In vitro	Good biocompatibility over hCLC and mouse macrophage RAW26.7 cells Highest antibacterial activity from CS-FA Highest antioxidant activity from CS-CA Highest lipid peroxidation from CS-SA	Lee et al. (2014) [161]
CS quaternary ammonium derivatives-FA, -pCA, -SA	Functional groups conversion	Antioxidant and antitumor agent	In vitro	Enhanced antioxidant properties Antitumor activity over A549 cells Good biocompatibility with L929 cells	Li et al. (2020) [124]
CS-CA	Carbodiimide- mediated	Anticancer agent	In vitro	Enhanced antitumour and anti-invasive effects over CT26 colorectal carcinoma cells	Lee et al. (2013) [165]
CS-CA	Ascorbic acid/hydrogen peroxide redox pair	Hepatoprotective agent	In vitro/in vivo	In vitro lipid peroxidation activity In vivo enhancement of antioxidant enzymes Reduction of pro-inflammatory molecules	Park et al. (2017) [122]
CS-FA, CS-CA	Ascorbic acid/hydrogen peroxide redox pair	Antioxidant agent	In vitro/in vivo	Decreased thermal stability and crystallinity In vivo increase of antioxidant enzymes and decrease of malondialdehyde levels in D-galactose-induced ageing mice CS-CA with the highest in vitro and in vivo antioxidant activity	Liu et al. (2014) [166]

CS, chitosan; FA, ferulic acid; CA, caffeic acid; SA, sinapic acid; MIC, minimum inhibitory concentration; hCLC, human Chang liver cells.

The reported promising results in the different biomedical applications of CS–HCAs put forth a solid baseline to assume that these conjugates will continue to be explored in the context of many other health issues. However, variations in reaction conditions, raw materials, and other particular factors make it difficult to compare products and establish a clear relationship between the employed conjugation strategy and the product’s biological activities. Thus, researchers are encouraged to carry out more in-depth studies to accurately characterize the CS–HCA conjugates.

## 6. Perspectives and Limitations

Both CS and HCAs have shown many health benefits due to their advantageous biological properties. These properties are enhanced when CS and HCAs are conjugated and give place to structured biomaterials with improved activities or act as superior active ingredients. Here, the reported results for some potential biomedical applications of CS–HCAs have been discussed. However, there are still many health benefits of CS and HCAs alone that have not been evaluated in their conjugated form. As for the biomedical applications that have been discussed in this review article, there is still some way to go. For example, the neuroprotective effects of CS–HCAs have been little studied but have shown promising results in injury models, which could be assessed for different neurological disorders. In addition, an increasing interest in the antimicrobial properties of these conjugates could emerge given the impending antibiotic resistance in human pathogens.

It is worth noting that most of the reviewed studies are preliminary and employ in vitro models where mechanical enhancement and specific biological activities can be established. The focus of these works is on the granted properties of the synthesized conjugates, leaving aside the structure–function relationship to understand how these properties can be modulated by specific modifications or varying the nature of the graft. In this regard, coupling reactions can still be optimized towards specific designs according to each application. Enzymatic coupling reactions could be a promising strategy for this purpose given their high specificity, but the production and reuse of enzymes must be improved first. Chemical reactions are currently the favorite strategy for CS–HCA synthesis; however, safety and sustainability concerns may leave room for improvement. An interesting approach could be done through green chemistry, where coupling reactions are assayed with an environmental, health, and safety perspective.

Additionally, the shortage of in vivo studies leads to the need of evaluating CS–HCAs within complex systems. These reasons open the opportunity of carrying out more in-depth studies to understand the molecular basis of CS and HCA conjugates, especially for biomedical applications. 

## Figures and Tables

**Table 1:** Chitosan-hydroxycinnamic acid conjugates in the delivery of therapeutic molecules.

Conjugate	Synthesis Method	Application	Study Type	Properties	Reference (Year)
CS-pCA	Carbodiimide-mediated	Encapsulation of essential oil	In vitro	Higher thermostability, antioxidant activity, and antitumoral effect	Kamal et al. (2021) [146]
CS-FA	Ascorbic acid/hydrogen peroxide redox pair	BSA loading	In vitro	Higher thermal stability, swelling ratio, and superior sustained release	Li et al. (2021) [144]
CS-*trans*FA, CS-CA, or CS-SA	Enzymatic (laccase)	Methylene blue loading	In vitro	CS-AC with higher swelling capacity CS-*trans*FA and CS-CA biocompatible with HEK293 cells Sustained release independent of the grafting substrate	Huber et al. (2017) [149]
CS-FA	Ascorbic acid/hydrogen peroxide redox pair	BSA microencapsulation	In vitro	Higher thermal stability, lower crystallinity, and enhanced sustained release	Li et al. (2017) [143]
CS-FA	Ascorbic acid/hydrogen peroxide redox pair	Thiamine and pyridoxine microencapsulation	In vitro/in vivo	Higher thermal stability of thiamine/pyridoxine, lower crystallinity on modified CS, and good encapsulation rate In vivo controlled release and reduced effective dose of thiamine/pyridoxine	Chatterjee et al. (2016) [145]
CS-CA co- polymerized with D-*b*-PEG	Carbodiimide- mediated	Doxorubicin nanoencapsulation	In vitro	Enhanced water solubility Cell internalization of nanoparticles and growth inhibition of doxorubicin-resistant CT26 cells	Lee et al. (2015) [148]
Thiolated CS-pCA	Carbodiimide-mediated	Piperine encapsulation	In vitro	Increased swelling capacity, mucoadhesion, and controlled release of hydrophobic piperine	Pengpong et al. (2014) [147]

CS, chitosan; pCA, p-coumaric acid; FA, ferulic acid; CA, caffeic acid; SA, sinapic acid; D-b-PEG, dextran-*b*-poly(ethyleneglycol).

## Data Availability

Not applicable.
